# Investigation of the change in marker geometry during respiration motion: a preliminary study for dynamic-multi-leaf real-time tumor tracking

**DOI:** 10.1186/1748-717X-7-218

**Published:** 2012-12-18

**Authors:** Rie Yamazaki, Seiko Nishioka, Hiroyuki Date, Hiroki Shirato, Takao Koike, Takeshi Nishioka

**Affiliations:** 1 Graduate School of Health Sciences, Hokkaido University, Sapporo, Japan; 2 Department of Radiology, NTT East Japan Sapporo Hospital, Sapporo, Japan; 3 Department of Biomedical Sciences and Engineering, Faculty of Health Science, Hokkaido University N12W5, Sapporo, Kita-ku, 060-0812, Japan; 4 Department of Radiology, Hokkaido University School of Medicine, Sapporo, Japan; 5 Department of Internal Medicine, NTT East Japan Sapporo Hospital, Sapporo, Japan

**Keywords:** Tumor tracking, Dynamic multi-leaf collimator, Lung cancer, Radiotherapy

## Abstract

**Background:**

The use of stereotactic body radiotherapy (SBRT) is rapidly increasing. Presently, the most accurate method uses fiducial markers implanted near the tumor. A shortcoming of this method is that the beams turn off during the majority of the respiratory cycle, resulting in a prolonged treatment time. Recent advances in collimation technology have enabled continuous irradiation to a moving tumor. However, the lung is a dynamic organ characterized by inhalation exhalation cycles, during which marker/tumor geometry may change (i.e., misalignment), resulting in under-dosing to the tumor.

**Findings:**

Eight patients with lung cancer who were candidates for stereotactic radiotherapy were examined with 4D high-resolution CT. As a marker surrogate, virtual bronchoscopy using the pulmonary artery (VBPA) was conducted. To detect possible marker/tumor misalignment during the respiration cycle, the distance between the peripheral bronchus, where a marker could be implanted, and the center of gravity of a tumor were calculated for each respiratory phase. When the respiration cycle was divided into 10 phases, the median value was significantly larger for the 30%-70% respiratory phases compared to that for the 10% respiratory phase (*P*<0.05, Mann–Whitney *U*-test).

**Conclusions:**

These results demonstrate that physiological aspect must be considered when continuous tumor tracking is applied to a moving tumor. To minimize an “additional” internal target volume (ITV) margin, a marker should be placed approximately 2.5 cm from the tumor.

## Findings

### Introduction

**Figure 1:**
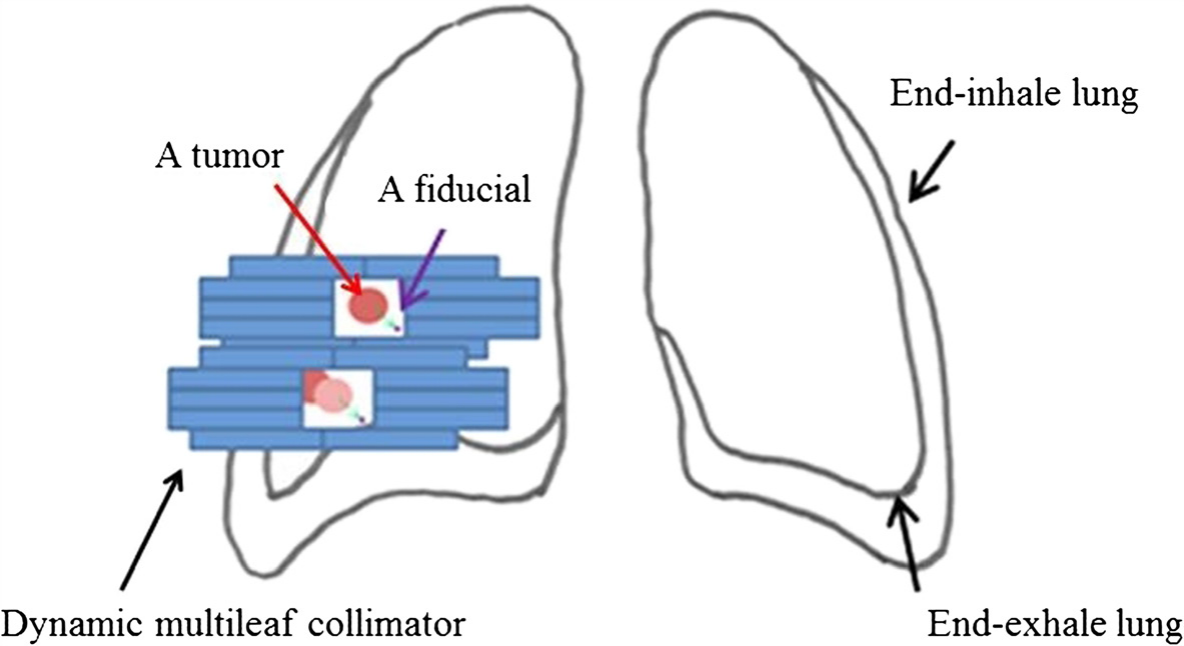
**The marker/tumor geometry change concept.** A possible fiducial/tumor misalignment at end-inhale (lower square). The pink circle is the expected correct tumor position. If misalignment occurs, the radiation dose to the tumor (painted in red) will be lower than planned.

Recent technological developments in radiation therapy, including body frames [1], infra-red light monitoring of chest wall movement to predict tumor location [2], and implanting fiducial markers (hereafter referred to simply as “markers”) for direct tumor targeting [3], have enabled high-dose radiation delivery to moving tumors, such as non-small cell lung carcinoma (NSCLC). With image guidance, most stage T1-2 NSCLC cases have become curable, with a local control rate of approximately 80% [4]. In our real-time tumor tracking radiotherapy system (RTRT), a beam becomes activated when markers fall into an end-exhale at 8 mm^3^[5]. End-exhale is suitable because it accounts for a relatively long time spent in a respiration cycle [5]. However, other phases are wasted, leading to prolonged treatment time (i.e., 30–40 min in our facility), and possible associated patient discomfort. Small but non-negligible skin doses are another concern [6]. Tumor tracking radiotherapy with a dynamic multi-leaf collimator (DMLC-RTRT) may help address these problems; experiments are ongoing, focusing primarily on a collimation system latency to follow tumor movement [7-9]. However, little research has been conducted on possible problems arising from marker/tumor geometry changes during respiration cycles. In this study, we discuss the possible concerns of DMLC-RTRT; the study concept is shown in Figure 1.

## Materials and methods

### Patients and imaging techniques

The present study included 8 patients with lung cancer who were candidates for stereotactic radiotherapy. Images were taken with a 64-Multi Detector-row Computed Tomography (MDCT) instrument (TOSHIBA, AquilionTSX-101A). The slice thickness was 2.0 mm and a respiration synchronization device (Anzai Medical AZ-733 V) was used. The scan was initiated after careful observation, ensuring that respiratory waves were stable on a monitoring screen [10]. Details about the 4D CT reconstruction procedure are described in our previous report [11]. Briefly, a respiratory-specific image was created from 0% (end-exhale) to 100% with 10% increments. This study strictly followed the guidelines of the Declaration of Helsinki and its amendments of 1983, 1989, 1996, as well as those of the internal ethics committee of our hospital. Median patient age was 78 years (range: 48–87 years); the male/female ratio was 5:3. Six patients had adenocarcinoma, one had squamous carcinoma, and one had metastatic carcinoma. All patients were node-negative; 4 patients had stage T1 tumors, 3 patients had stage T2 tumors, and 1 patient was not staged because the tumor was metastatic.

### Image interpretation

To detect possible marker/tumor misalignment during the respiration cycle, the distance between the peripheral bronchus, where a marker could be implanted, and the center of gravity of a tumor were calculated for each respiratory phase. Anatomical detection was performed on a Digital Imaging and Communications in Medicine (DICOM) viewer with the free software ImageJ [12] (pixel size, 0.64 mm^2^). To improve anatomical detection, images were enlarged two times.

#### The center of gravity of a tumor

**Figure 2:**
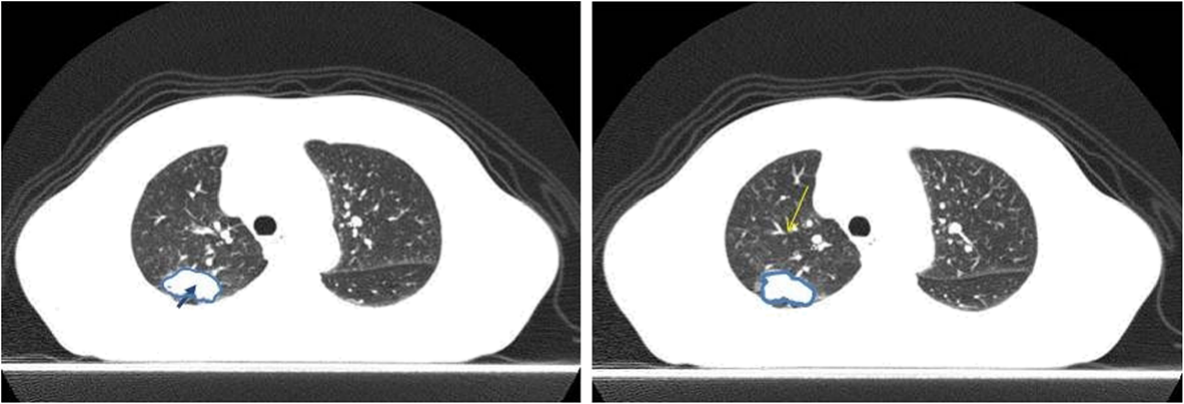
**The distal bronchus and the center of gravity of the tumor.** Note that the distal bronchus is not visible, but can be detected by VBPA (yellow arrow). The center of gravity (blue arrow) was estimated by delineating the tumor contours (blue) at each respiratory phase on ImageJ.

Two board-certified radiologists contoured the tumor at each respiratory phase image. The center of gravity of the tumor was calculated from those images, and this point was used as a reference for the marker/tumor distance (Figure 2).

#### Peripheral bronchi as surrogates for markers

The peripheral bronchi were used as surrogates for markers. The advantage of using bronchi is that they can be selected at any distance from the center of gravity of a tumor. The peripheral bronchi mentioned here have a diameter ≤ 1.5 mm. Such distal bronchi were often not visible, but could be detected by virtual bronchoscopy using the pulmonary artery (VBPA) [13]. This technique was originally developed in our facility to better detect peripheral bronchi (with a diameter ≤ 1.5 mm) to improve biopsy success rates. We have found this to be a very good tool for identification of the small bronchi required in this study. The bifurcations of the peripheral arteries were chosen as surrogate points for bronchi, based on the principle that the bronchi always run together with the pulmonary arteries.

On average, the inter-observer difference both for the center of gravity and bronchus-surrogate point was 0.4 mm (maximum, 0.8 mm). Contouring and distal-bronchus detection using VBPA were performed independently by the two board-certified radiologists. The averages of the coordinates of the center of gravity and the distal bronchus were used for misalignment evaluation.

## Results

**Figure 3:**
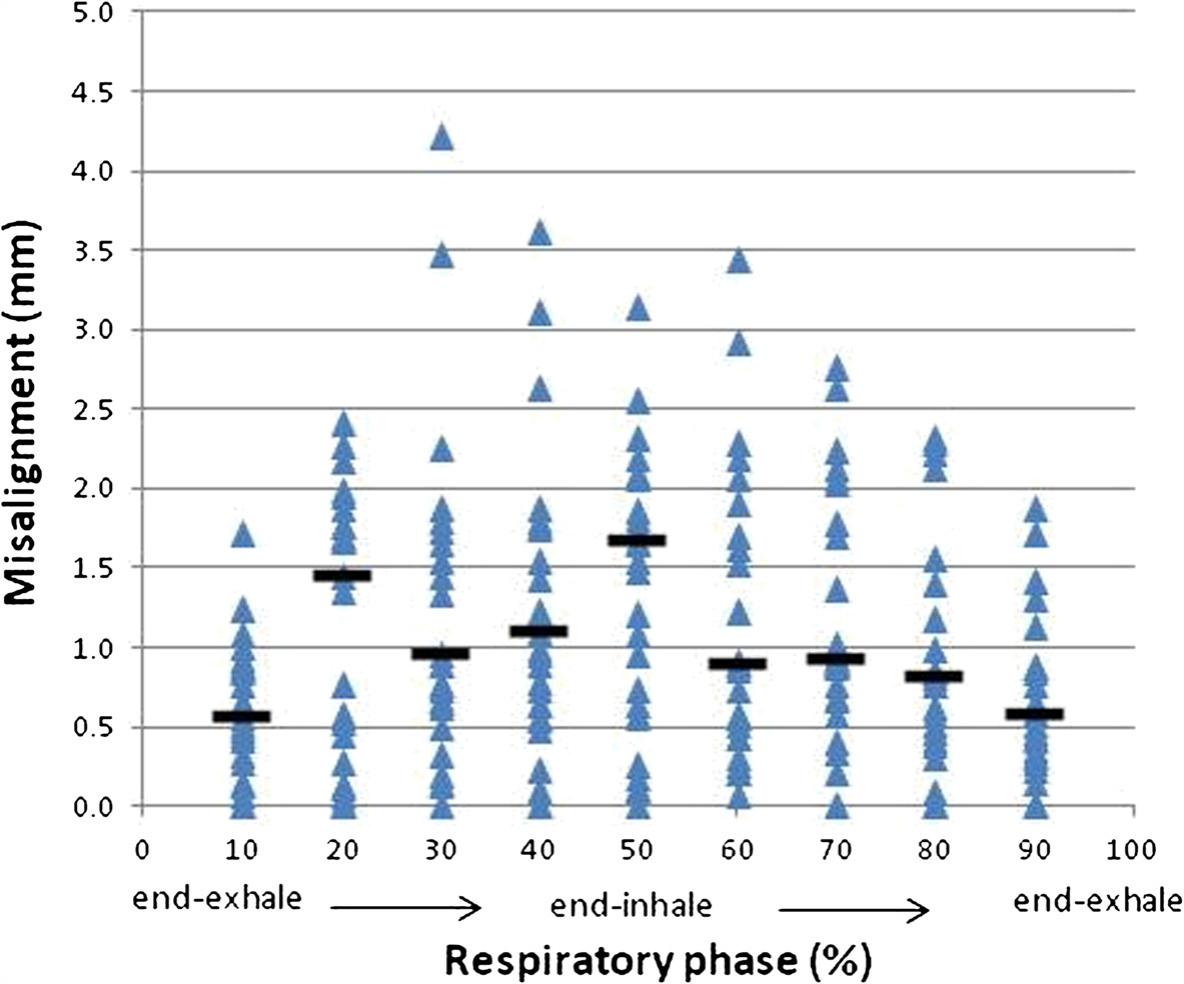
**The marker/tumor misalignments as a function of respiratory phase for the 25 distances.** A statistically significant difference in misalignment was observed between the 30%-70% respiratory phases and the 10% respiratory phase (*P*<0.05, Mann–Whitney *U*-test). The horizontal line represents the median value for each respiratory phase.

**Figure 4:**
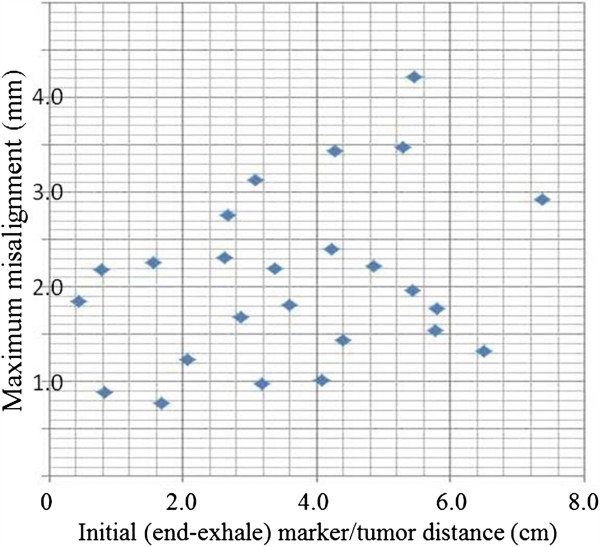
**Maximum misalignments.** Maximum misalignments are shown with respect to initial (i.e., at end-exhale) marker/tumor geometry. Note that the misalignment of ≥2.5 mm did not occur in cases with an initial marker/tumor distance of ≤2.5 cm.

An example of marker/tumor detection is shown in Figure 2. A total of 25 distances (i.e., an average of 3 bronchi per patient) were calculated. The median misalignment at each phase among the 25 distances is shown in Figure 3. A statistically significant differences in misalignment was observed between the 30%-70% respiratory phases and the 10% respiratory phase (*P*<0.05, Mann–Whitney *U*-test). When the initial marker/tumor distances were divided into two categories―a) < 3 cm (n=7) and b) ≥ 3 cm (n=18)―the misalignment was larger for the latter group at the 40%-70% respiratory phases (*P*<0.05, Mann–Whitney *U*-test). The maximum misalignment with respect to the initial marker/tumor geometry for each patient is plotted in Figure 4. Although no statistically significant difference between tumor/marker distance and misalignment was observed, it is important to be aware that misalignment can be >2.5 mm in cases with a marker/tumor distance of 2.5 cm.

## Discussion

Misalignment may be greater for tumors located at the base of the lung, where motion is greater. In fact, a German research group demonstrated a large lung architecture movement above the diaphragm [14]; however, no tumors were located above the diaphragm in the present study. Special attention would be required for proper marker implantation of such tumors. These results imply that it might be safer to increase the internal target volume (ITV) margin in DMLC-RTRT compared to that of “near-static” RTRT.

Considering the steep dose–response curve for stereotactic radiotherapy of the lung, careful treatment planning is required when DMLC-RTRT is administered to a case in which misalignment is >2.5 cm. Even a slight misalignment could increase the chance of marginal recurrence. For example, reducing the dose from 48 Gy/4 fr to 40 Gy/4 fr resulted in lower tumor control [5]. Currently, several markers are often inserted for tumor tracking, potentially increasing treatment accuracy [15]. A group from the University of Texas Health Science Center performed a phantom DMLC-RTRT study, in which the displacement between the DMLC beam isocenter and the marker therein ranged from 0.5-1.5 mm. These researchers also reported that DMLC-RTRT reduces the mean surrounding tissue dose by 43% when compared to three-dimensional conformal radiation therapy (3DCRT). The percentage of lung volume receiving at least 20 Gy (V20) therein will be reduced from 28% to 18%, and the dose to 20% of the lung volume (D20) from 35.2 Gy to 15.0 Gy [16]. Another phantom study evaluating a MLC for 4D radiotherapy in the lung demonstrated that an MLC latency period of 570 ms and DMLC-RTRT will be of clinical use, provided that the respiration-related isocenter misalignment is within 2 mm [17]. This group also performed a series of experiments using a prototype of kilovoltage/megavoltage DMLC-RTRT and reported that the root-mean-square errors in the beam-target alignment ranged from 3.1-7.6 mm without tracking and were reduced to <1.5 mm with tracking, except during the model-building period (6 s) [18].

All of the studies mentioned above were phantom-based; however, physiological factors must also be considered during clinical use. Targeting a moving lung tumor is a state-of-the-art technology. Such advanced engineering is most likely not available in most regional hospitals. A practical solution is to use the “mid-ventilation” method, in which an image from a set of 4D scans represents the tumor in its time-averaged position over the respiratory cycle (the mid-ventilation CT scan) [19]. There is a consensus that an inter-fractional isocenter shift is within 6 mm [20]. Cone-beam CT may be a practical tool to determine the isocenter. So far, physical characteristics have been intensively investigated for precise high-dose radiotherapy. However, biological aspects may also need to be considered for this type of radiotherapy. We have conducted irradiation experiments in cancer cells that mimicked high-dose/fraction radiotherapy. Cells that survived a 10-Gy radiation dose exhibited higher motility and invasiveness in a three-dimensional collagen gel [21]. Thus, such cells may implement mechanisms to try to escape from the high-dose irradiation field. A new concept of a “biologically targeted volume” may therefore be needed for precise high-dose radiotherapy.

## Conclusion

DMLC-RTRT is a rapidly developing-technique with promising data from leading institutions. However, when applying these techniques in a clinical setting, physiological aspects must be considered, as shown in the present study. It might be safer to place a marker approximately 2.5 cm from the tumor, to minimize an “additional” ITV margin. Minimizing the ITV margin may be beneficial for patients with multiple small lesions or proximally located tumors that are associated with a high risk of complications [22]. The value of 2.5 cm is a rough estimate, and further study with more patients is warranted to confirm the present findings.

## Abbreviations

SBRT: Stereotactic body radiotherapy; VBPA: Virtual bronchoscopy using pulmonary artery; ITV: Internal target volume; MDCT: Multi Detector-row Computed Tomography; RTRT: Real-time tumor tracking radiotherapy; DMLC: Dynamic multi-leaf collimator; D20: The dose to 20% of the lung volume; V20: The percentage of lung volume receiving at least 20 Gy; 3DCRT: Three-dimensional conformal radiation therapy.

## Competing interest

The authors report no conflicts of interest with respect to this work.

## Authors’ contributions

RY: study design, SN: data acquisition, HD: constructive critique, HS: constructive critique, TK: constructive critique, TN: lead study investigator. All authors read and approved the final manuscript.
